# Reverse optic capture in cataract surgery: techniques, indications, and outcomes

**DOI:** 10.3389/fopht.2026.1760370

**Published:** 2026-03-11

**Authors:** Eric W. Lai, Daniel Henick, Brian Debroff

**Affiliations:** Department of Ophthalmology and Visual Science, Yale School of Medicine, New Haven, CT, United States

**Keywords:** anterior capsulotomy, capsular support, IOL capture, IOL centration, IOL rotational stability, lens capsule, negative dysphotopsia, reverse optic capture

## Abstract

Reverse optic capture (ROC) is a surgical technique in which the optic of an intraocular lens (IOL) is positioned anterior to the anterior capsulotomy opening, while the haptics remain within the capsular bag. Described originally as a method to secure fixation in cases of posterior capsular rupture, ROC has since evolved as a surgical maneuver to address fixation of an IOL in the setting of a not-intact posterior capsule, toric IOL rotation, treatment of negative dysphotopsia, and correction of unanticipated postoperative hyperopia. This review highlights the development, indications, and outcomes of ROC, as well as its advantages, limitations, and future directions. Additionally, we present a case of ROC utilized to treat negative dysphotopsia. As surgical techniques and IOL technologies continues to evolve, further prospective investigation will help clarify the role of ROC in achieving stable and predictable outcomes in complex cataract and refractive surgery.

## 1 Introduction

Advances in cataract surgery techniques, particularly the development of phacoemulsification and continuous curvilinear capsulorhexis (CCC) have enabled precise intraocular lens (IOL) implantation and stable long-term results (1). Additional progress in IOL technology has expanded the range of available lens designs, from monofocal lenses to toric, multifocal, and extended depth-of-focus (EDOF) optics, each with unique demands for centration, rotational stability, and capsular support (2).

Despite these advances, intraoperative and postoperative challenges remain – posterior capsule rupture, zonular instability, IOL malrotation, and visual disturbances such as negative dysphotopsia can compromise visual outcomes (3). Various fixation techniques have since been developed, including various optic capture, sulcus placement, scleral fixation, and iris fixation to manage these complex scenarios (4).

Gimbel and DeBroff presented the versatility of optic capture in 2004 as a means to enhance IOL centration and reduce posterior capsule opacification (PCO) (5). In rhexis fixation IOL capture, the optic is pushed posteriorly through the anterior capsule rim, while the haptics remain in the sulcus. This configuration promotes IOL stability, centration, and helps to prevent lens epithelial cell migration or postoperative refractive surprise (**Table 1**) (6).

**Table 1:** Comparison of IOL fixation configurations.

Technique	Optic position	Haptic position	Capsular relationship	Indication
In-the-bag fixation	Posterior to anterior capsule, fully within capsular bag	Haptics within capsular bag	Optic and haptics enclosed by capsule	Routine cataract surgery
Rhexis optic capture	Posterior to anterior capsulotomy opening	Haptics in sulcus	Optics captured behind anterior capsule rim	Posterior capsule compromise; prevention of PCO; centration support
Reverse optic capture	Anterior to anterior capsulotomy opening	Haptics within capsular bag	Optic engaged anterior to capsule rim, haptics secured in bag	Posterior capsule compromise; toric IOL rotation; negative dysphotopsia

In contrast, the technique of reverse optic capture (ROC) is used when an IOL is placed within the capsular bag, and the IOL optic is placed anteriorly through an intact anterior capsulotomy opening. The versatility of ROC lies in its ability to address a wide spectrum of intraoperative complications and postoperative challenges that can compromise IOL stability and visual outcomes. Although originally conceived as a rescue maneuver following posterior capsule rupture, its use today has expanded to manage IOL malrotation, negative dysphotopsia, and cases of capsular or zonular compromise (7). This review and case presentation reviews the clinical and surgical indications, the technique, the outcomes, and the limitations of reverse optic capture.

## 2 Methods

A systematic literature was conducted using the PubMed database without date restrictions. PubMed was selected as the primary database given its comprehensive indexing of peer-reviewed medical and ophthalmic literature. Search terms included “reverse optic capture”, “optic capture”, “IOL capture”, and “IOL management”, and “negative dysphotopsia”, queried individually and in combination. Reference lists of relevant articles were manually reviewed to identify additional studies.

Peer-reviewed, PubMed-indexed articles discussing ROC in human subjects were included encompassing all types of study design. Studies were excluded if they did not specifically address ROC or describe unrelated optic capture techniques. Titles and abstracts were screened for relevance, and full texts were reviewed when necessary. Due to heterogeneity in study designs and outcomes, findings were synthesized descriptively without quantitative meta-analysis.

In addition to the literature review, a single illustrative case of ROC performed for the treatment of negative dysphotopsia was included to further elucidate its clinical application. In accordance with Yale University’s institutional ethics policy, institutional review board (IRB) approval was waived for this single-patient case report. Informed consent for publication was obtained, and all clinical information was de-identified in compliance with HIPAA privacy regulations.

## 3 Clinical and surgical indications

### 3.1 Posterior capsular rupture

Posterior capsule rupture has the potential to make IOL placement more difficult, destabilize the intended refractive outcome, and complicate IOL fixation (8). When the integrity of the capsular bag is compromised, intraoperative options include placement of a three-piece IOL in the sulcus, anterior chamber IOL, iris-fixated IOL, or a scleral-fixated IOL. Each carries potential risks, including IOL decentration, corneal endothelial damage, chronic inflammation, cystoid macular edema (CME), and retinal tear or detachment (9). ROC provides an alternative option while preserving IOL fixation, avoiding sulcus-related complications, and maintaining refractive planning.

This technique is useful when a capsule tear occurs or is first noticed after placement of the IOL in the capsular bag. When a one-piece IOL is injected through a small incision and released into the capsular bag, the surgeon may first recognize a posterior capsular tear that extends upon IOL placement in-the-bag. In addition, a posterior capsular tear can occur after the IOL is in the bag. Finally, a surgeon may not recognize a posterior capsular tear until the IOL is in the capsule. In order to prevent subsequent IOL dislocation prior to the technique of reverse optic capture, the surgeon would need to remove the IOL from the ruptured bag, cut or fold it, and remove and replace it through the incision. Such manipulation often would necessitate an anterior vitrectomy and require sulcus placement of a three-piece IOL. With the technique of ROC, the one-piece IOL does not need to be explanted and replaced but rather secured by placing the IOL optic anteriorly through the capsulotomy opening (10, 11). This does require an anterior capsulotomy opening at least 1 mm smaller than the optic diameter of the IOL to achieve stable fixation. Jason et al. reported a series of 16 eyes of patients with ROC in the setting of posterior capsule rupture that achieved comparable results to a group of uneventful phacoemulsification procedures (10).

ROC can also be used when a posterior capsule rupture is recognized prior to IOL insertion. Jones et al. described using ROC in the setting of a PC tear to avoid having to place a sulcus IOL (7). This technique requires special care to avoid IOL posterior dislocation (see technique section). Finally, ROC can be utilized in cases when repositioning posteriorly dislocated IOLs. The vitreoretinal surgeon performing a pars plana vitrectomy can bring the IOL forward and by capturing the IOL optic anteriorly through an intact anterior capsulotomy opening, can achieve stable fixation.

In such a manner, all these methods of ROC lead to an intentional positioning of the IOL optic anterior to the intact capsulotomy opening while retaining the haptics in the capsular bag which leverages the anterior capsule rim for IOL stability (12).

### 3.2 Preventing IOL rotation when placing a toric IOL for correction of astigmatism

When treating astigmatism, the precise alignment of toric IOLs is critical to achieving the intended refractive correction, as even small degrees of rotation can significantly diminish astigmatic correction. Malrotation of greater than 30 degrees essentially nullifies the toric effect (13). While options such as IOL repositioning, lens exchange, or scleral fixation are available, they can be technically challenging and risk further destabilization of the capsular bag and zonular support. ROC offers a less invasive option as a long-term stabilizing strategy for toric IOLs prone to rotation (12). By positioning the IOL optic outside the capsular bag, the optic is mechanically constrained, which may enhance rotational stability and reduce the risk of unintended rotation. This may be particularly effective when placing toric IOLs in patients with large capsular bags (12). Reverse optic capture may be utilized to secure the IOL either at the time of initial IOL placement or at the time of IOL repositioning or IOL exchange due to clinically significant IOL rotation (12).

### 3.3 Treating negative dysphotopsia

Postoperative visual phenomenon has been well documented and difficult to address after cataract surgery. Negative dysphotopsia can be a debilitating phenomenon characterized by a temporal crescent-shaped shadow in the peripheral visual field. There are multiple proposed etiologies for negative dysphotopsia, one of the most accepted is that the interaction between the anterior capsule edge and the sharp edge of modern IOL optics, particularly small square-edge IOL designs, produces an arc of blocked light at the nasal retina (14). ROC has emerged as a potential intervention for negative dysphotopsia. By repositioning the optic anterior to the anterior capsule margin, the theory is that the optical edge contributing to the shadowing effect is displaced, which may alleviate the visual phenomenon (15). Masket et al. reported successful resolution of negative dysphotopsia in patients undergoing secondary ROC, highlighting its value not only as a treatment modality but also as a potential preventive strategy in at-risk patients (16).

### 3.4 Correction of residual hyperopia after in-the-bag IOL placement

Residual hyperopia can occur after even the most careful pre-operative measurements. Refractive surprises can have greater consequences when implanting multifocal IOL’s as the residual refractive result can lead to unhappy patient visual results. Akaishi et al. presented a series of 16 eyes that underwent a secondary procedure of ROC as a method to correct residual hyperopia after in-the-bag placement of a multifocal IOL (17). By effectively moving the IOL anteriorly with ROC, a mean hyperopic correction of 1.09 diopters was achieved (17).

### 3.5 Achieving IOL stability in cases of zonular weakness

In eyes with zonular weakness, such as those with traumatic injury, pseudoexfoliation, or collagen-vascular disease, achieving stable IOL fixation can be challenging. While capsular tension rings and other supportive devices improve stability, they may not be sufficient in cases with significant compromise (18). ROC may provide an additional layer of stabilization by anchoring the optic against the anterior capsule rim. When combined with capsular support devices, it may potentially improve IOL centration and reduce the risk of tilt or decentration (19).

## 4 Technique and variations

Successful employment of ROC requires a well-centered, continuous curvilinear capsulorhexis of appropriate size, one that is at least one millimeter smaller than the optic diameter to allow secure capture. A large or irregular CCC risks optic decentration or loss of capture in which the central optic falls back into the capsular bag. Several variations of the original ROC technique have been described. These modifications were developed to address specific anatomic challenges, optimize stability, and expand the clinical applicability of ROC across a wider range of surgical scenarios (5).

In traditional reverse optic capture, the lens is initially injected or inserted into the capsular bag in standard fashion. For the optic manipulation portion, a lens manipulator or spatula can be used to gently elevate the optic edge anterior to the CCC margin. The lens can then be finely adjusted to ensure that both haptics remain secure within the capsular bag while the optic is held anteriorly by the CCC rim. It is important during this step to confirm the IOL optic is well centered without any iris chafing (7). Intraoperatively, one can be certain that the intraocular lens has successfully been placed in reverse optic capture because there will be characteristic bending of the anterior capsulotomy where the optic exits the capsular bag.

ROC is more difficult when placing a one-piece IOL in the setting of a known rupture of the posterior capsule. This may be performed to avoid placing a sulcus IOL or also in the case where a surgeon would like to place a stabilized one-piece toric IOL even in the setting of a PC tear. The surgeon must use extreme care to avoid dislocating the IOL into the vitreous when performing such a maneuver and special techniques are necessary to the stabilize the IOL prior to capture to avoid dislocation. Jones published his technique of injecting the one-piece IOL anterior to the capsule and then carefully placing the IOL haptics posterior to the anterior capsulotomy while supporting the IOL optic with a spatula or Kuglen hook (7). Gimbel described a technique which he called the haptic tuck modification. This technique involves initially placing the IOL in the sulcus with the toric marks aligned with the corneal marks. Then, each haptic is separately tucked through the capsulotomy opening, leaving the optic edge above the capsule (20).

Additionally, the introduction of femtosecond laser technology into cataract surgery has provided opportunities to refine and ensure stable reverse optic capture. A precise, reproducible, and perfectly centered capsulotomy created by the femtosecond laser offers an ideal platform for optic capture. Most importantly, consistency in capsulotomy diameter and centration reduces the risk of asymmetric capture or optic decentration (21). Femtosecond laser-assisted ROC may therefore be particularly advantageous in cases involving premium IOLs, where centration is critical to optical performance. Although real world clinical data are lacking, early reports suggest that laser-created capsulorhexis can improve predictability and reproducibility of ROC (22).

## 5 Case presentation

To demonstrate the utility of the reverse optic capture (ROC) technique, herein we describe a case in which a patient underwent intraocular lens repositioning for management of negative dysphotopsia after cataract surgery.

A 76-year-old man with dry eyes, angular blepharitis, and bilateral cataracts underwent sequential cataract surgery (OD then OS). Preoperatively, best corrected visual acuity (BCVA) was 20/200 OD with 2+ nuclear sclerosis and 3+ posterior subcapsular cataract, and 20/30 OS with 2+ nuclear sclerosis. Dilation was 7 mm, and there was no pseudoexfoliation, phacodonesis, or trauma OU. During right-eye surgery, two clock hours of zonular weakness were managed with a capsular tension ring, and a toric IOL was implanted, resulting in 20/20 vision on postoperative day 1. Left-eye surgery was uncomplicated with toric IOL placement at 168°.

Postoperatively, BCVA was 20/20, but the patient reported a temporal crescent consistent with negative dysphotopsia OS. The IOL remained well-positioned in the capsular bag with no subluxation or tilt, and OCT macula revealed only a minimal epiretinal membrane not involving the fovea. The anterior capsulotomy was approximately 5.5 mm (0.5 mm smaller than the optic diameter) and the IOL haptics were aligned at the intended axis OS. Symptoms persisted over several months despite stable findings. Treatment options—including observation, IOL repositioning with reverse optic capture, and IOL exchange—were discussed. The patient elected for IOL repositioning using the reverse optic capture technique OS, understanding the risk of regression due to his large capsulorhexis. The procedure went without complication, and the optic was successfully repositioned over the anterior capsulorhexis while maintaining the intended toric axis of alignment.

**Figure 1:**
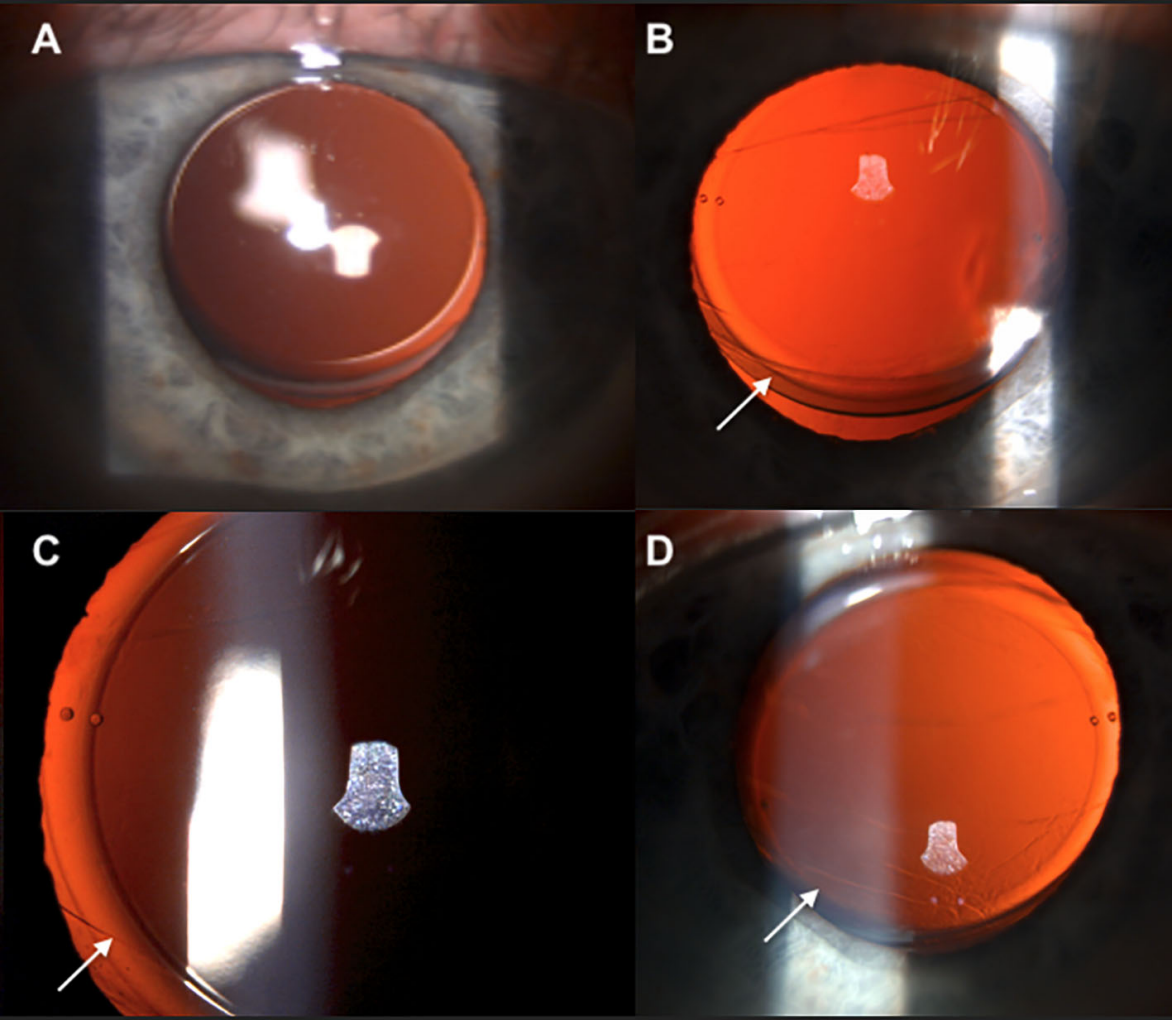
Preoperative IOL in traditional capsular bag position **(A)**. Postoperative day 1 after reverse optic capture lens repositioning with “bending” appearance of the capsulorhexis, indicating correct IOL position [**(B, C)** arrows]. Postoperative month 1, the bend in capsulorhexis is less apparent, indicating loss of reverse optic capture [**(D)** arrow].

On postoperative day 1 after IOL repositioning with ROC, the patient had mild visual decline of 20/40 in the left eye with 1+ corneal edema but resolved temporal shadow. The IOL was well aligned at the intended axis and noted to be in reverse optic capture position (**Figure 1**). At 1 week, vision improved to 20/20, and he felt his temporal crescent had significantly decreased. By 1 month, the IOL was found to have returned to the capsular bag, losing its reverse optic capture positioning and at the same time, the patient noticed the negative dysphotopsia had returned, but was not bothered enough to consider additional intervention. This case demonstrates improvement of negative dysphotopsia with ROC, return of negative dysphotopsia after loss of ROC, and emphasizes the need for a capsule at least one millimeter in diameter smaller than the IOL optic diameter for long term capture stabilization.

## 6 Discussion

The emergence of ROC as both a rescue maneuver and a deliberate surgical strategy has expanded the options available to cataract surgeons during cases of compromised capsular support or postoperative complications. The growing body of literature, though largely composed of case reports and small series, consistently demonstrates favorable outcomes. In the setting of posterior capsule rupture, Jones et al. reported that more than 90% of eyes achieved 20/25 or better vision when managed with ROC of single-piece acrylic IOLs. Importantly, IOL centration and refractive outcomes were preserved, demonstrating that ROC can serve as a viable alternative to sulcus or anterior chamber IOL implantation (7).

In cases of toric IOL instability, Gimbel et al. demonstrated the use of ROC as a successful strategy to stabilize toric IOL. In their report, a toric IOL that had rotated more than 70 degrees postoperatively was repositioned and stabilized using ROC with haptic tuck technique, after which the IOL maintained proper alignment for over two years. This case highlights the potential of ROC to provide durable, long-term rotational stability in toric IOLs prone to postoperative malrotation (20).

Additionally, Masket et al. demonstrated that repositioning the optic anterior to the capsulotomy margin alleviated temporal dark shadows in most patients, with secondary ROC procedures successfully eliminating symptoms in cases resistant to conservative management (16). Moreover, ROC may serve as a preventive measure in patients at high risk of IOL instability or those with anatomic predispositions to negative dysphoptosia. In our case, although ROC was able to resolve negative dysphotopsia, the large capsulorhexis size likely led to regression of ROC and recurrence of negative dysphotopsia. Use of femtosecond laser assisted cataract surgery, can help ensure predictable capsulotomy size, centration, and perfect circularity, which can help ensure stable IOL fixation with ROC.

Despite its advantages, ROC is not without limitations. The most significant constraint is its dependence on an intact and properly sized continuous curvilinear capsulorhexis. A large, eccentric, or irregular CCC may preclude stable optic capture and limit its applicability in eyes with extensive capsular damage (23).

Additionally, by positioning the optic more anteriorly, ROC may result in a modest myopic shift compared to standard in-the-bag placement due to the modified effective lens position. This could be beneficial in patients with a postoperative unintended hyperopic shift. In patients without hyperopia, however, ROC may lead to a myopic shift that could be detrimental especially in patients with extended depth of field IOL’s or multifocal IOL’s (24).

Potential complications, although reported infrequently, include iris chafing, pigment dispersion, and elevated intraocular pressure, particularly when the optic is asymmetrically captured or when excessive anterior vaulting results in intermittent iris contact. Chronic iris-optic contact can lead to pigment release, anterior chamber inflammation, and in severe cases, UGH syndrome (25). These risks may be more pronounced in rescue settings – such as cases with compromised capsular support – where zonular instability or irregular capsulorhexis configuration can make symmetric capture more technically challenging. In contrast, planned primary ROC in eyes with intact capsular support may allow for more controlled and symmetric optic positioning, potentially mitigating these risks. Some studies have also suggested a potential for earlier PCO, possibly related to reduced optic-capsule overlap and altered capsular bend formation; however, long-term comparative data remain limited (26).

Finally, the current literature on ROC is largely composed of retrospective series, case reports, and noncomparative studies, often with small sample sizes and limited long-term follow-up. Heterogeneity in surgical technique, IOL design, and clinical indications further limits direct comparison across studies, and randomized comparative trials remain scarce. Although reported outcomes are generally favorable, the overall level of evidence remain low to moderate, and larger prospective, multicenter studies and real0world outcome data are needed to more definitively establish the efficacy, reproducibility, and long-term safety profile of ROC. Incorporating ROC into surgical practice therefore requires careful patient selection, meticulous attention to capsulorhexis size and centration, and an understanding of its refractive and anatomical considerations. Within these parameters, ROC represents a valuable addition to the cataract surgeon’s armamentarium, offering an alternative between standard in-the-bag fixation and more complex surgical approaches. As IOL technologies continue to evolve, further study will help clarify the role of ROC in achieving stable and predictable patient outcomes.

## 7 Conclusion

Reverse optic capture (ROC) has evolved from a rescue maneuver for posterior capsule rupture into a versatile technique for stabilizing intraocular lenses in challenging scenarios such as toric IOL malrotation, negative dysphotopsia, unintended postoperative hyperopia, and zonular compromise. By engaging the anterior capsule margin, ROC preserves posterior chamber fixation while providing secure and predictable refractive outcomes. Although current evidence is limited, reported results demonstrate favorable visual and refractive outcomes. Large prospective studies are warranted to confirm long-term efficacy and safety as ROC emerges as a valuable tool in modern cataract and refractive surgery.
